# Non-melanoma skin cancer of the external auditory canal: long-term outcomes of a tertiary head and neck unit

**DOI:** 10.1007/s00405-025-09274-3

**Published:** 2025-02-20

**Authors:** Eleanor Apthorp, Rebecca Lam, Rupert Obholzer, Jean-Pierre Jeannon, Richard Oakley, Aleix Rovira

**Affiliations:** 1 GKT School of Medical Education, King’s College London, Guy’s Campus, Great Maze Pond, London, SE1 1UL UK; 2 Department of Otolaryngology and Head and Neck Surgery, Guy’s and St Thomas’ NHS Foundation Trust, Great Maze Pond, London, SE1 9RT UK; 3 Guy’s and St Thomas’ NHS Foundation Trust, Westminster Bridge Road, London, SE1 7EH UK

**Keywords:** External auditory canal carcinoma, Lateral temporal bone resection

## Abstract

**Purpose:**

External auditory canal (EAC) skin cancer is often diagnosed at advanced stages, leading to poor survival outcomes. Our study aims to describe disease characteristics, treatments and outcomes of patients with EAC cancer, increasing understanding of the management of this rare disease.

**Methods:**

Retrospective, observational study including patients with non-melanoma EAC skin cancer treated at Guy’s and St Thomas’ Head and Neck Unit from 2012 to 2021, with follow-up until October 2023. Patient with EAC or auricular primaries extending into the EAC were included. Demographic, histopathological, and surgical data were obtained from electronic records.

**Results:**

Thirty-eight patients were included, 86.8% treated with curative intent. The median follow-up was 49.9 months. One, three, and five-year overall survival for patients treated curatively were 100%, 96.9% and 75.3%, respectively, versus 40.0%, 0.0% and 0.0% for palliative. 68.4% had advanced disease (Pittsburgh staging, III: 18.4%, IV: 50.0%). 39.5% were treated after recurrent or persistent disease. Histological subtypes included squamous cell carcinoma (60.5%), basal cell carcinoma (26.3%) and others (13.2%). Among those treated surgically (*n* = 31), 74.2% underwent lateral temporal bone resection and 29.0% wide local excision. 83.9% had parotidectomy, neck dissection or both. 51.6% received post-operative radiotherapy/chemoradiotherapy. Advanced stage was significantly associated with reduced overall survival (*p* = 0.05) but not disease-free survival (*p* = 0.25). No primary site features, regional metastasis (*p* = 0.63), direct parotid invasion (*p* = 0.71) or age (*p* = 0.15) significantly impacted survival.

**Conclusion:**

According to the good outcomes reported, this study suggests lowering the threshold for radical treatment may improve outcomes for patients with potentially poor prognostic features.

## Introduction

External auditory canal carcinoma (EACC) is incredibly rare, accounting for only 0.2% of all head and neck cancers [1, 2]. It often remains undiagnosed until advanced stages due to its subtle and benign-like symptoms which mimic other otologic conditions, such as otitis externa. Risk factors for EACC include chronic otitis media, prior radiation therapy and chronic ear disease, although the role of the later remains controversial due to limited evidence [2, 3]. EACC is most commonly squamous cell carcinoma (SCC) with other histological subtypes observed including basal cell carcinoma (BCC) or adenoid cystic carcinoma (AdCC) among others [4]. Diagnosis includes physical examination, biopsy and imaging.

The prognosis of EACC is highly dependent on the stage at diagnosis, with 5-year survival rates for advanced disease (stage III-IV SCC) as low as 40.4%, compared to 84.5% for early-stage (stage I-II SCC) cases [5]. Treatment often requires extensive surgical resection, followed by radiotherapy and sometimes chemotherapy [6]. Treatment of EACC is particularly challenging due to the complex anatomy of the temporal bone and the proximity of dura, cranial nerves and blood vessels. The proximity and potential invasion of these structures makes achieving clear surgical margins difficult, which adversely impacts survival outcomes [4]. Patterns of tumour spread including facial nerve involvement, bone erosion, direct parotid invasion (DPI) and parotid and cervical lymph node invasion (LNI) are associated with poorer outcomes [2, 7, 8]. Understanding of clinical, histological and radiographic presentation of EACC is essential to its accurate diagnosis and management, to optimize therapeutic guidelines and ultimately improve its prognosis.

This study aims to retrospectively analyse patients with EACC to characterise disease presentation, treatments approach and long-term outcomes, with the goal of improving management strategies and prognosis.

## Methods

### Study population

This retrospective, observational study included all patients consecutively treated for EACC at the head and neck cancer unit at Guy’s and St Thomas’ Hospital with EACC from January 2012 until December 2021. Patients were identified from a prospectively maintained non-melanoma skin cancer database. Patients with tumours affecting the EAC, including tumours that originated on the pinna, pre-auricular and post-auricular areas were included for analysis. Patients were excluded if complete clinical, histological, or imaging data were unavailable for analysis.

### Data collection

Perioperative, histological and follow up data were collected from both electronic medical records and paper-based notes. Information regarding patient demographics, clinical presentation, facial nerve function, tumour staging, and treatment history was recorded. Tumour status was classified as primary, persistent (< 6 months from previous treatment), or recurrent (> 6 months from previous treatment). Preoperative histological characteristics, including tumour subtype and degree of differentiation, were noted. Radiological assessments, including Computed Tomography (CT), Magnetic Resonance Imaging (MRI), Ultrasound (US), and Positron Emission Tomography (PET), were utilized to evaluate tumour extent and regional/distant metastasis. Postoperative histology included assessments of LNI, DPI, perineural invasion, lymphovascular invasion, bone involvement, intracranial invasion, and dural involvement. Tumours were classified according to the modified Pittsburgh T-staging system, derived from the Arriaga staging system [9, 10].

As determined by the NHS Health Research Authority decision tool, this study qualified as a service evaluation and did not require formal ethical approval. Approval was granted as an audit under project number 14,378 at Guy’s and St Thomas’ Hospital. Patient confidentiality was ensured, and all data were anonymized before analysis.

### Treatment protocol

All patients included were discussed in the Head and Neck multidisciplinary team (MDT) meeting including head and neck surgeons, medical oncologists, radiologists, pathologists and allied healthcare professionals. Surgical resection was the preferred curative treatment, with radiotherapy (RT) reserved for patients with early-stage disease and comorbidities precluding surgery. Palliative care was offered to those unfit for curative treatment or those who refused it. Dural and intracranial involvement on imaging resulted in non-surgical palliative treatment.

The extent of surgical resection was determined by tumour invasion, ranging from limited EAC wide local excision (WLE) to lateral temporal bone resection (LTBR), in cases with osseous EAC invasion. When facial palsy or nerve involvement was identified, the facial nerve was sacrificed and immediately reconstructed with static or dynamic reconstruction decides on individual basis. Pinna resection was performed when necessary to achieve clear surgical margins. Parotidectomy and neck dissection were performed in radiologically N + patients or N0 patients with advanced stage (T3/T4), except for cases with BCC, as decided by the MDT. Post-operative RT was administered to all patients with AdCC and in patients with perineural invasion, close or positive margins, extracapsular nodal involvement and/or with lymph node involvement (> 3 cm). Concurrent systemic therapy was considered in cases with positive margins or/and extranodal extension (ENE) based on extrapolation of guidelines of mucosal cancers in the absence of prospective comparative studies [11].

### Statistical analysis

Data were analysed using R Studio (version 4.1.2). Kaplan-Meier survival curves were used to estimate overall survival (OS) and disease-free survival (DFS). Log-rank tests compared survival between subgroups, while Cox proportional hazards regression evaluated the impact of tumour characteristics on survival, adjusting for confounders. Statistical significance was defined as *p* < 0.05. OS was defined as the time from diagnosis (biopsy date) to death from any cause, and DFS as the time from diagnosis to recurrence or death from any cause.

## Results

### Patient and disease

Thirty-eight patients with EACC were treated at our head and neck surgery unit from 2012 to 2021. Demographic characteristics, treatment intention, histological type and tumour staging are summarised in Table 1. Median age at diagnosis was 67 (33–90) with a predominance of male sex (73.7%). Of note, 39.5% of patients had tumours that were either persistent or recurrent after initial surgery elsewhere. According to the modified Pittsburgh TNM classification, definitive tumour staging considering clinical and pathology data was distributed as follows: early stage in 12 cases (31.6%; stage I — 13.2%; stage II — 18.4%) and advanced stage in 26 cases (68.4%; stage III — 18.4%; stage IV — 50.0%).

**Table 1:** A summary of the demographics of patients, tumour histology and staging of patients with advanced external auditory canal carcinoma (*n* = 38). Clinical stage is assessed according to the Pittsburgh Staging System, determined by post-operative histopathology in patients who underwent surgery and radiological and biopsy results in those treated palliatively. Key: G1 (well differentiated), G2 (moderately differentiated), G3 (poorly differentiated), N0 (no nodal involvement), N+ (pathologically confirmed positive nodes), M0 (no distant metastases), M+ (presence of distant metastases)

	N = 38
Median follow up	49.9 months (range = 3.0-205.1)
*Sex*	
Female	10 (26.3%)
Male	28 (73.7%)
*Age*	
Median age at diagnosis	
All patients	67 years (IQR = 17, range = 33–90)
Palliative (n = 5)	81 years (range = 67–90)
Curative (n = 33)	63 years (range = 33–89)
*Treatment Intention*	
Curative	33 (86.8%)
Palliative	5 (13.2%)
*Tumour status*	
Primary	23 (60.5%)
Persistence	5 (13.2%)
Recurrence	10 (26.3%)
*Histology*	
Squamous Cell Carcinoma	23 (60.5%)
G1	2 (8.7%)
G2	5 (21.7%)
G3	9 (39.1%)
Unknown	7 (30.4%)
Basal Cell Carcinoma	10 (26.3%)
Adenoid Cystic Carcinoma	3 (7.9%)
Adenocarcinoma	1 (2.6%)
Trichoblastic Carcinoma	1 (2.6%)
*Stage*	
Size of tumour	
T1	4
T2	9
T3	11
T4	14
Lymph node invasion (LNI)	
Cervical LNI	8
Parotid LNI	1
No LNI	14
No neck dissection	15
Distant metastases	
M0	37
M+	1
Pittsburgh Stage	
I	5 (13.2%)
II	7 (18.4%)
III	7 (18.4%)
IV	19 (50.0%)

### Treatment

A total of 33 patients (86.8%) received curative-intent treatment. Of these, 31 underwent surgery, while 2 were treated with radical radiotherapy (RT) due to being medically unfit for surgery. Among those who underwent surgery, 54.8% (17/31) required additional oncological treatment, with 12 patients receiving postoperative radiotherapy (PORT) and 5 receiving postoperative chemoradiotherapy (POCRT). Surgical and adjuvant treatments, along with postoperative complications and recurrence rates, are summarized in Table 2. Five patients (13.2%) were treated with palliative intent after discussion in the multidisciplinary team (MDT).

Among the 31 surgically treated patients, 74.2% (23/31) underwent LTBR with or without neck dissection and/or parotidectomy. Other procedures included WLE and total or partial pinnectomy. The decision to perform neck dissection and its extent was made on a case-by-case basis, considering tumour stage, histology, preoperative nodal status, and reconstruction needs.

Five patients (16.1%) experienced postoperative complications. Two complications were related to primary surgery: one patient developed meatal stenosis following wide local excision for recurrent BCC, leading to cholesteatoma, and required further surgery; another patient had a cerebrospinal fluid leak requiring surgical repair after LTBR with parotidectomy and neck dissection. Two patients developed free-flap collections. One patient experienced significant osteoradionecrosis of the temporal bone following PORT.

### Histology

SCC was the most common histological subtype, found in 60.5% (20/33) of patients, followed by BCC in 26.3% (10/33), AdCC in 7.9% (3/33), and rare subtypes including trichoblastic carcinoma (2.6%) and adenocarcinoma (2.6%). Of the 23 patients who underwent neck dissection, 8 (34.8%) had positive cervical LNI. LNI was most common in SCC (5/8), with other cases occurring in patients with BCC (1), AdCC (1), and adenocarcinoma (1). The most frequent site of lymph node metastasis in SCC was level IIB (4/5 patients). Seven patients had ENE of lymph node metastases. Three patients who underwent parotidectomy had DPI, and one had parotid LNI.

Postoperative pathology revealed that 20 patients (64.5%) had clear margins, 6 (19.4%) had close margins (< 5 mm), and 2 (6.5%) had positive margins. Data on margin status were missing for 3 patients (9.7%).

Of the patients treated curatively, 11 had perineural invasion (PNI). 14 patients had bone invasion. 6 patients in total had facial nerve paralysis, 5 of these patients were treated curatively.

**Table 2:** A summary of the pathological results, adjuvant therapies and post-operative reconstruction and complications in patients who underwent surgery (*n* = 31) and the recurrence prevalence of patients treated with curative intent (*n* = 33)

	N = 31
*Surgery*	
LTBR (total or subtotal)	24 (77.4% of 31 patients who had surgery)
Total (including temporomandibular joint (TMJ) resection)	22 (2)
Subtotal	2
Wide local excision (WLE)	7 (22.6% of 31 patients who had surgery)
Concurrent neck dissection (ND)	24 (77.4% of 31 patients who had surgery)
	23 (74.2% of patients who had surgery)
Concurrent parotidectomy (PD)	1
Partial PD	21
Total PD	
*Pathology results*	
Cervical lymph node invasion	8 (of 24 who had ND) (34.8%)
Levels affected	No. patients with levels affected
Level Ia	0/8
Level Ib	1/8
Level IIa	5/8
Level IIb	6/8
Level III	1/8
Level IV	0/8
Level V	0/8
Parotid node metastasis	1 (of 23 who had PD) (4.3%)
Direct parotid invasion	3 (of 23 who had PD) (13.0%)
*Margins*	
Clear	20 (64.5%)
Close (< 5mm)	6 (20.0%)
Positive	2 (6.7%)
Missing	3 (10.0%)
*Adjuvant therapy*	
Post-operative radiotherapy (PORT)	12 (38.7% of 31 surgical patients)
Post-operative chemoradiotherapy (POCRT)	5 (16.1% of 31 surgical patients)
Total adjuvant therapies	17 (54.8% of 31 surgical patients)
*Post-operative reconstruction*	
Free flap reconstruction (FFR)	13 (41.9% of 31 surgical patients)
*Post-operative complications*	
Relating to primary surgery	2 (6.5% of 31 surgical patients)
Related to reconstruction	2 (15.4% of 13 who had FFR)
Related to adjuvant therapy	1 (5.9% of 17 patients who had PORT)
*Recurrence*	4 (12.1% of 33 patients treated with curative intent)

### Survival outcomes

**Fig. 1:**
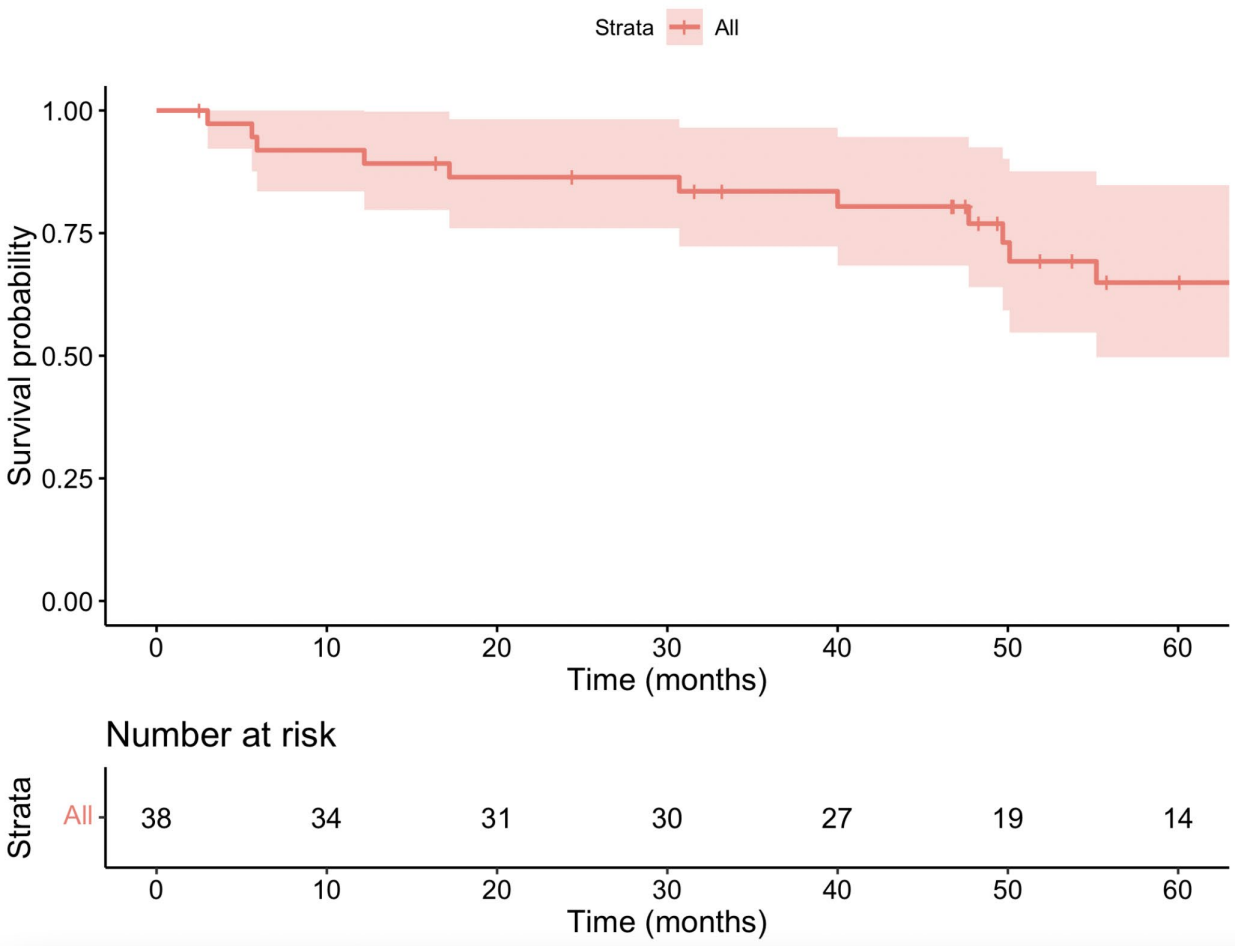
Kaplan-Meier survival analysis of overall survival of patients with external auditory canal carcinoma from date of diagnosis to last follow-up (time (months)) (*n* = 38)

**Fig. 2:**
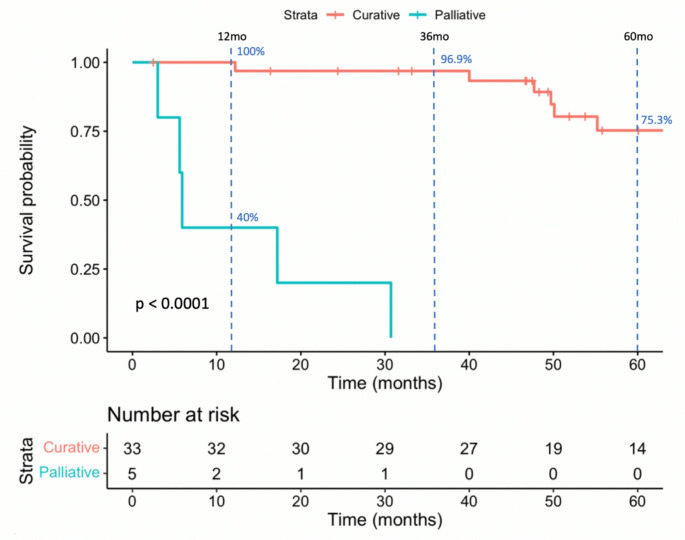
5-year overall survival in patients with external auditory canal carcinoma according to treatment intention. (*n* = 38, Log Rank *p* < 0.0001). Estimated 12, 36 and 60 month (1, 3 and 5 year respectively) overall survival of patients treated curatively (*n* = 33) and palliatively (*n* = 5) is displayed

The median follow-up for all patients was 49.9 months (range 3.0–205.1 months). Figure 1 displays the survival outcomes for all patients in the study. Among patients treated curatively, the median follow-up was 53.8 months. During the study period, 4 patients (12.1%) experienced recurrence, with 3 having local recurrence and 1 regional recurrence. Of these, 3 patients died, and 1 patient was disease-free at the end of follow-up after undergoing further excision for local recurrence. The estimated 5-year OS for patients treated with curative intent was 75.3%, and the estimated 5-year DFS was 71.1%. In contrast, among the 5 patients treated palliatively, the 1-year OS was 40.0%, and no patient survived beyond 3 years. Figure 2 displays these results.

**Fig. 3:**
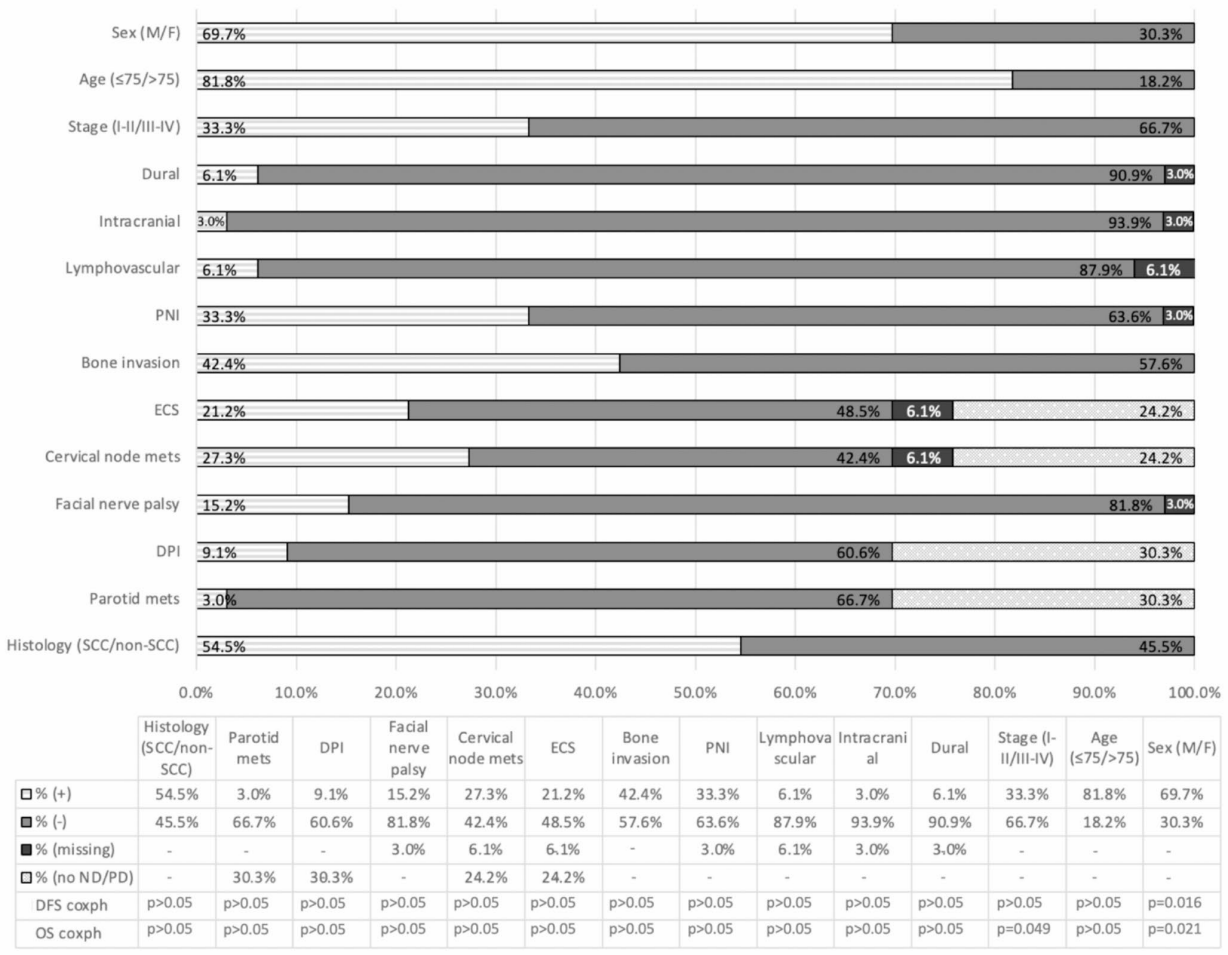
A summary of the prevalence of primary site features in patients treated with curative intent and their correlation with disease-free survival (DFS) and overall survival (OS), using Cox proportional hazard analysis. (*n* = 33) Patient demographics and primary site features are displayed on the y-axis and the proportions of each feature in the sample are displayed using bars, with the % along the x-axis. The key at the bottom displays the raw data and explains which shading corresponds with feature presence, absence or missing data. In the case presence of extracapsular spread, cervical node metastasis and parotid invasion, shading indicates whether these features are absent, present or if the patient did not have a neck dissection or parotidectomy. The disease-free survival and overall survival Cox proportional hazard p-values are displayed in the table

The impact of primary tumour characteristics on OS and DFS was analysed using Cox proportional hazard models (Fig. 3). Advanced tumour stage (stage III-IV) was significantly associated with reduced OS (*p* = 0.049). Male sex was also associated with poorer OS and DFS (*p* = 0.021 and 0.016). No other primary tumour characteristics, including histological subtype, lymph node invasion, or bone invasion, were significantly associated with survival outcomes (*p* > 0.05). Table 3 provides a summary of all patients in the cohort.

**Table 3:** A summary of key demographic and tumour information for all patients with external auditory canal carcinoma (*n* = 38). Staging is according to Pittsburgh staging system. Lymph node invasion is only assessed in patients who underwent neck dissection and parotidectomy. Key: M (male), F (female), SCC (squamous cell carcinoma), BCC (basal cell carcinoma), AdCC (adenoid cystic carcinoma), TC (trichoblastic carcinoma), G1 (well differentiated), G2 (moderately differentiated), G3 (poorly differentiated), C (curative intent), P (palliative intent), S (surgery), R (radiotherapy), C (chemotherapy), 0 (no treatment), N/A (not available), LNI (lymph node invasion), N+ (positive nodes), N0 (no positive nodes), Y (yes), N (no), FN (facial nerve status), I (intact facial nerve), Pa (paretic or paralysed facial nerve), A (alive), D (deceased)

Patient	Demographic	Histology	Intention	Treatment	Stage	LNI	FN	Recurrence	Status
1	74 F	SCC (G3)	C	S + R	IV	N+	I	N	A
2	59 M	AdCC	C	S + R	IV	N+	I	N	D
3	71 M	BCC	C	S	I	N0	I	N	A
4	74 F	SCC	C	S	I	N0	Pa	N	A
5	51 M	BCC	C	S	II	N0	I	N	A
6	89 M	SCC (G2)	C	S	III	-	I	N	D
7	57 M	SCC (G1)	C	S	III	N0	I	N	A
8	67 M	SCC (G3)	C	S + R	III	N0	I	N	A
9	67 M	TC	C	S	II	N0	I	N	A
10	52 M	SCC	C	S + R	III	N0	Pa	N	A
11	71 M	SCC (G2)	C	S	II	N0	I	N	A
12	33 F	BCC	C	S	III	-	I	N	A
13	57 F	SCC (G3)	C	S + R	IV	N0	I	N	D
14	86 M	SCC (G2)	C	S	I	N0	I	N	D
15	79 F	BCC	C	S + R + C	IV	N0	I	Y	D
16	57 M	BCC	C	S	II	-	I	N	A
17	55 M	BCC	C	S	I	-	I	N	A
18	60 F	Adenocarcinoma	C	S + R	IV	N+	Pa	N	A
19	68 F	SCC	C	R	IV	-	I	N	A
20	81 M	SCC (G3)	C	S + R	II	N0	I	N	A
21	55 F	AdCC	C	S + R	IV	N0	Pa	N	A
22	57 M	SCC (G3)	C	S + R + C	IV	N0	Pa	Y	D
23	56 M	SCC (G3)	C	S + R + C	IV	N+	I	N	A
24	72 F	SCC (G3)	C	S	I	N0	I	N	A
25	61 M	BCC	C	R	IV	-	I	Y	D
26	79 M	SCC (G2)	C	S + R	IV	N0	I	N	D
27	76 M	BCC	C	R	III	-	I	N	A
28	58 M	SCC (G3)	C	S + R	III	N+	I	Y	A
29	70 M	BCC	C	S	IV	N0	I	N	A
30	72 M	BCC	C	S	IV	N+	I	N	D
31	63 M	SCC	C	S + R + C	IV	N+	I	N	A
32	63 F	AdCC	C	S + R	II	N0	I	N	A
33	61 M	SCC (G3)	C	S + R + C	IV	N+	I	N	A
34	75 M	SCC	P	R	IV	-	I	-	D
35	81 M	SCC	P	0	IV	-	I	-	D
36	87 M	SCC	P	R	IV	-	I	-	D
37	67 M	SCC (G1)	P	0	IV	-	Pa	-	D
38	90 M	SCC (G2)	P	R	III	-	I	-	D

## Discussion

EACC is a rare malignancy typically associated with poor outcomes, particularly in advanced stages. However, our cohort showed encouraging survival rates for patients treated with curative intent, indicating that aggressive surgical intervention combined with adjuvant therapies can lead to improved outcomes. The 5-year overall survival (OS) rate in our study supports these findings, with high survival rates observed even in advanced-stage patients.

### Demographics

The median age of diagnosis in our cohort was 67, aligning with existing literature, which indicates that EACC primarily presents in the 7th decade of life, often related to cumulative sun exposure and immunosuppression [2–5, 12, 13]. Consistent with previous studies, the majority of patients were male, likely due to delayed presentation in men, who may seek medical help later than women [14]. This is reflected in our cohort, where 68.4% of patients presented with advanced disease requiring referral to a tertiary centre.

Age at diagnosis significantly impacted treatment approaches and outcomes in our cohort. Patients treated palliatively had a median age of 80 (range 67–90), compared to a median of 65.2 (range 33–89) for those treated curatively. A robust pre-operative MDT assessment is crucial in identifying patients who can tolerate aggressive treatments and optimizing those with frailty or significant comorbidities for surgery and PORT [15, 16]. At our institution, the Perioperative Medicine for Older People (POPS) team played a vital role in pre-surgical optimization [15]. The POPS team provide a comprehensive, holistic assessment to inform decision making including data such as morbidity and mortality scores. This approach is essential to ensure patients undergoing extensive, mutilating surgeries are optimised to reduce the risk of peri-operative complications and to maximise the chances of total disease clearance.

### Staging and histology

EACC is often diagnosed at advanced stages due to its rarity and nonspecific symptoms [3]. This was apparent in our cohort, with patients often having symptoms for more than 12 months before biopsy. Advanced stage (III and IV) SCC is strongly associated with worse outcomes, therefore early recognition and diagnosis would be significant in improving outcomes [12, 17, 18]. In our cohort, patients with stage III-IV disease had significantly reduced overall survival compared to stage I-II (5-year OS: 90.9% vs. 72.7%, *p* = 0.049). Other studies report varying survival outcomes, with Chi et al. [18] reporting a 5-year OS of 0% for advanced-stage SCC treated with extensive surgery and PORT, whilst Goto et al. [19] reported similar outcomes to our study with 5-year OS of 100% and 61.7% for patients with stage III and IV tumours respectively. Our study showed improved outcomes, possibly due to differences in patient selection, comorbidities, or surgical techniques.

Previous studies have described good accuracy of the Pittsburgh staging system, with a correlation between more advanced stages and worse prognosis [7, 12, 20, 21]. Using Pittsburgh staging, our cohort included 12 (31.6%) patients diagnosed as stage I-II [9]. The larger proportion of advanced staged disease is a consequence of the nature of the disease as already described and due to our cohort being treated by a tertiary H&N unit, with most patients with stage I and II disease being treated by local otolaryngologists or dermatologists. In our cohort, pre-operative radiological staging and extensive histopathology results were available for patients who underwent major resections. However, non-surgical patients were staged clinically and radiologically only. In the 18 patients with both histopathological and radiological staging available, the two modalities reported the same tumour stage in 66.7% of cases, suggesting some consistency despite the varying techniques. A study compared radiological and pathological staging in EACC and found some correlation between pre-operative CT scans and post-operative pathology when assessing tumour invasion [22]. There is always discrepancy between clinical, histological and radiological staging in any tumour site. However, due to the anatomical complexity and complexity of the staging systems themselves, the variation is even more relevant in EACC. Radiological assessment by sub-specialist head and neck radiologists is essential to guide surgical approach and tumour staging plays a useful role in treatment planning both pre- and post-operatively.

SCC is the most common histology for H&N skin cancers, as seen in our cohort [4, 18, 23, 24]. There was no significant difference in OS or DFS between histological subtypes despite other studies finding significantly reduced survival in patients with SCC compared to other histologies [12, 25]. A systematic review including 437 patients with EAC SCC reported overall 5-year survival of 53.0%, whilst our study found estimated overall 5-year survival of 72.7% for all histologies and 83.3% for SCC alone in patients treated curatively [5]. The advancement of skull-based microsurgery and progress in imaging techniques may further contribute our promising outcomes [21]. Further sub-group analysis according to histology is essential. In particular, this would inform adaptation of the current staging system for EACC as it currently does not account for different histologies which potentially incur different prognoses [9].

### Nodal spread

The incidence of LNI in EAC SCC is estimated to be 10–23% [5], but in our cohort, LNI was observed in 29.0% of surgical patients, likely due to the advanced nature of the disease. Gidley et al. described the LNI rate to be higher in periauricular and external ear tumours (27.5% and 26.9% respectively) compared to EACC tumours (8.0%), potentially explaining the rate of LNI in our cohort [26]. Most of the nodal involvement was localized to level II, with 8 patients presenting with cervical LNI and 1 with parotid LNI. While LNI is typically a poor prognostic factor [3, 21, 26], our findings did not show a significant impact on OS or DFS. This contrasts with previous studies but aligns with Qiu et al. [20], who found no significant relationship between LNI and survival in middle ear SCC [3, 20, 21]. They concluded T-classification to have a stronger prognostic role than nodal status in 214 patients with middle ear SCC [20]. This is further supported by Ito et al. who proposed extent of bone erosion to be the most significant prognostic factor in temporal bone SCC [27]. Our results, supported by these studies, suggest tumour invasion depth and location may be more prognostically relevant than nodal status due to increased difficulty acquiring clear margins [20, 27]. Findings such as these have led to debate about reassessing the Pittsburgh staging system to place greater emphasis on T-status, whilst still considering N and M status [20].

One patient with BCC had LNI. This is an interesting entity as BCC characteristically has a very low metastasis rate, ranging from 0.0028 to 0.55%, rendering our case extremely rare [28]. The patient maintained DFS for four years following LTBR, ND and PD, however passed away from a different cancer. There is very limited literature regarding LNI in BCC making this a unique observation therefore neck dissection and parotidectomy are not recommended for clinically/radiologically N0 disease with this histology type, however can be indicated in advanced stage (III-IV) disease or when free flap reconstruction is needed.

In H&N cancer, it is established that prophylactic neck dissection is indicated when the risk of occult neck disease is above 20% [29]. However, no clear indication with regards to EACC exists, resulting in treatment policies according to the centre [30]. With the reported number of cervical LNI and the likely need for neck exploration for vessel preparation when the free flap reconstruction is needed, the authors support a low threshold for performing prophylactic neck dissection.

DPI was not significantly associated with reduced overall survival in our cohort, with the rate of invasion being similar to other studies [12]. Some authors report an association between DPI and poor prognosis, however others propose that other factors, including PNI and LNI, impact survival more significantly than DPI [31, 32]. Despite this, it is widely established that carrying out PD is highly advised, especially in T3 and T4 tumours as PD carries low mortality rates, outweighing the risks of further disease spread [31, 32]. Our study supports this, with all patients with DPI (*n* = 3) or parotid LNI (*n* = 1) maintaining remission and observing no post-operative complications in all 23 patients who underwent PD.

### Facial nerve palsy

Facial nerve involvement, often a marker of advanced disease, is associated with worse outcomes due to the risk of perineural invasion leading to intracranial spread and automatically resulting in a stage IV classification according to the modified Pittsburgh staging [8, 9, 10, 12]. However, in our cohort, facial nerve palsy did not significantly impact OS or DFS (Fig. 3). This may be due to the use of aggressive surgical techniques, where facial nerve sacrifice was performed to achieve clear margins and prevent further dissemination. Despite the poor prognosis associated with facial nerve invasion, radical resection should be considered to maximize disease remission. In this situation, expert reconstructive surgeons are required. This is particularly important in the rehabilitation of the facial nerve which is a niche reconstructive area of expertise. Poor prognosis associated with advanced stage tumours is often taken as a reason not to operate, even in otherwise well patients. Our data supports a more aggressive approach.

TMJ resection was carried out in two patients, an aggressive approach to achieve maximal disease clearance in advance tumours. Goto et al. [19] carried out similarly extensive surgical management, with six of their surgical 30 patients undergoing TMJ resection. Their survival outcomes reflect ours, supporting our recommendation for more aggressive surgical management of EACC [19]. We concur that extended LTBR, including the TMJ, is required and recommended to achieve clear margins in locally advanced tumours, however the increased operative time and intraoperative blood loss must be considered when assessing fitness for surgery [19].

### Limitations

This study’s retrospective design and small sample size limit the strength of its conclusions. However, given the rarity of EACC, prospective studies are likely unfeasible. Our study, the first of its kind in the UK since Bibas et al. [33], provides valuable insights into a rare disease and its management at a high-volume tertiary centre. Future research should focus on optimizing adjuvant therapies and investigating novel treatments, such as cetuximab, to reduce the need for mutilating surgeries while maintaining survival outcomes [34]. Larger scale analysis into which clinical and tumour factors play the most significant prognostic role is essential to further refine the Pittsburgh staging system, a contentious issue.

## Conclusion

This study adds valuable understanding to the current knowledge surrounding EACC, carried out at single centre tertiary H&N unit. A high level of surgical expertise has been developed likely contributing to the good results reported, supporting the principle of further centralisation for this rare disease. LNI, parotid invasion and facial nerve palsy had no significant impact on prognosis, however advanced stage significantly reduced OS. This indicates that although surgery and aggressive PORT are mutilating, they significantly impact life expectancy, allowing many patients to live cancer-free with low recurrence rates. Compared to similar sized studies, our cohort had better five-year survival, providing encouraging results. According to the good outcomes reported, even with advanced stage diagnosis, this study suggests lowering the threshold for radical treatment may improve outcomes for patients with potentially poor prognostic features.
